# Clinical benefits and risks of remote patient monitoring: an overview and assessment of methodological rigour of systematic reviews for selected patient groups

**DOI:** 10.1186/s12913-025-12292-w

**Published:** 2025-01-23

**Authors:** Constanze Wartenberg, Helen Elden, Malte Frerichs, Lennart L Jivegård, Kajsa Magnusson, Georgios Mourtzinis, Ola Nyström, Kajsa Quitz, Helen Sjöland, Therese Svanberg, Helena Vallo Hult

**Affiliations:** 1 Region Västra Götaland, HTA-Centrum, Sahlgrenska University Hospital, Gothenburg, Sweden; 2 Department of Obstetrics and Gynecology, Region Västra Götaland, Sahlgrenska University Hospital, Gothenburg, Sweden; 3 Department of Respiratory Medicine and Allergology, Region Västra Götaland, COPD Center, Sahlgrenska University Hospital, Gothenburg, Sweden; 4 Region Västra Götaland, Sahlgrenska University Hospital, Medical library, Gothenburg, Sweden; 5 Department of Molecular and Clinical Medicine, Institute of Medicine, Sahlgrenska Academy, University of Gothenburg, Gothenburg, Sweden; 6 Department of Medicine and Emergency Mölndal, Region Västra Götaland, Sahlgrenska University Hospital, Gothenburg, Sweden; 7 Department of Medicine Geriatrics and Emergency Medicine, Region Västra Götaland, Sahlgrenska University Hospital/Östra Hospital, Gothenburg, Sweden; 8 Region Västra Götaland, Egenmonitoreringscenter, Närhälsan, Gothenburg, Sweden; 9 Department of Planning and Development, Region Västra Götaland, NU Hospital Group, Trollhättan, Sweden; 10 Department of Informatics, School of Business, Economics and IT, University West, Trollhättan, Sweden

**Keywords:** Remote patient monitoring, Remote home monitoring, Telemonitoring, Systematic review

## Abstract

**Background:**

Remote patient monitoring implies continuous follow-up of health-related parameters of patients outside healthcare facilities. Patients share health-related data with their healthcare unit and obtain feedback (which may be automatically generated if data are within a predefined range). The goals of remote patient monitoring are improvements for patients and reduced healthcare costs.

The aim of this paper is to provide an overview of systematic reviews regarding remote patient monitoring for selected patient groups currently considered for the introduction of remote patient monitoring in Region Västra Götaland, Sweden. The selected sixteen patient groups were: patients with asthma, chronic obstructive pulmonary disease, children and adolescents with complex needs, children and adolescents with cystic fibrosis, children and adolescents with periodic fever, elderly patients with multiple diseases, patients with eye diseases, heart failure, haematological disease, hypertension, inflammatory bowel disease, neurorehabilitation, Parkinson’s disease, psoriasis, sleep apnea, and specialist maternity care. Outcomes considered in this overview were patient-relevant clinical benefits as well as risks.

**Methods:**

A literature search for systematic reviews of clinical trials on remote patient monitoring in the selected patient groups was conducted by two information specialists, followed by assessment of relevance by a team of clinical and methodological experts in Region Västra Götaland, Sweden. The methodological rigour of identified systematic reviews was assessed using QUICKSTAR – a tool for stepwise appraisal of systematic reviews. In a QUICKSTAR assessment, a level of at least five is considered a prerequisite for reliable conclusions regarding the question at issue.

**Results:**

The literature search resulted in 4,049 hits, of which 84 SRs were considered relevant for the question at issue. A QUICKSTAR level of at least five was reached by 13 (15%) of the relevant systematic reviews. Some patient benefit of remote patient monitoring was reported for five patient groups (asthma, chronic obstructive lung disease, heart failure, hypertension, and elderly patients with multiple diseases). For four patient groups (children with complex needs, children with cystic fibrosis, specialist maternity care, and sleep apnea), systematic reviews of adequate quality concluded that scientific evidence on clinical patient benefits of remote monitoring is very limited. For seven patient groups, no systematic reviews of sufficient quality were identified.

**Conclusion:**

Clinical benefits and risks of remote patient monitoring as a replacement for, or in addition to, standard of care compared to standard of care (face-to-face visits) are poorly studied for most of the selected patient groups based on systematic reviews of acceptable quality. Patient-relevant clinical benefits are limited or impossible to evaluate for most diagnoses based on currently available scientific information. Possible clinical risks and costs are poorly studied.

**Supplementary Information:**

The online version contains supplementary material available at 10.1186/s12913-025-12292-w.

## Summary box

*What is already known on this topic* – Development and implementation of remote patient monitoring is increasing in many diagnosis groups, and clinical effects are described in a growing number of systematic overviews.

*What this study adds* – This overview identifies systematic reviews on remote patient monitoring in 16 patient groups with focus on clinical outcomes and provides a systematic assessment of the methodological rigour of these systematic reviews.

*How this study might affect research, practice or policy* – This overview highlights the need for further controlled studies of patient-relevant clinical benefits and risks of remote patient monitoring and the need for methodological rigour when performing systematic reviews. A distinction is needed between using remote patient monitoring as a replacement and offering remote patient monitoring in addition to standard of care face-to-face visits. Especially if remote patient monitoring is to replace face-to-face visits and to increase healthcare efficiency, further research on patient-relevant clinical benefits, patient risks, and the use of healthcare resources compared to standard of care is needed.

## Background

Worldwide, healthcare is facing significant challenges due to factors such as an ageing population, increased survival rates from life-threatening diseases, and the growing burden of chronic illnesses [1–4]. These challenges have strained healthcare resources and led to the need for innovative solutions to prioritise and optimise limited resources while managing rising healthcare costs [5, 6]. Digital health broadly refers to the use of information and communication technologies to deliver healthcare services remotely and help people monitor and manage their health and wellness [7]. It includes various heterogeneous technologies that allow information exchange, patient education, consultations, and delivery of care in multiple forms, tied together by remote healthcare resources delivery [8, 9]. Digital health technology, such as mobile apps, wearable devices, and sensors, is seen as a solution to provide sustainable care, reduce costs, and improve medical quality, accessibility, and quality of life [6, 10]. Furthermore, these technologies are increasingly explored as tools for supporting patient decision-making and self-care [11, 12].

Remote patient monitoring allows patients to monitor their health and medical conditions outside traditional healthcare facilities. It personalises recommendations and treatment based on health data generated by patients outside conventional clinical settings, thus transforming healthcare delivery from synchronous face-to-face to remote and asynchronous [13]. Though recent technological advances have addressed some challenges, issues such as data privacy, security, management, scalability, regulations, and interoperability persist [9, 13]. Prior research has mainly focused on remote patient monitoring within chronic conditions like diabetes, where it is expected to yield the most significant benefits [13].

Currently, remote patient monitoring is developed in many areas of healthcare, aiming for improved access to and quality of healthcare and increased efficiency of healthcare providers. In this paper, remote patient monitoring is defined as follows: patients located outside healthcare facilities (e.g., at home) measure or record specified health-related parameters that are digitally transferred to a healthcare unit, which in turn provides the patient with feedback on the reported data. The feedback may be automatically generated if the patient’s data are within a predefined range.

In the region Västra Götaland in Sweden, the introduction of remote patient monitoring is considered in healthcare units providing care to patients with asthma, chronic obstructive pulmonary disease, children and adolescents with complex needs, children and adolescents with cystic fibrosis, children and adolescents with periodic fever, elderly patients with multiple diseases, patients with eye diseases, heart failure, haematological disease, hypertension, inflammatory bowel disease, neuro-rehabilitation, Parkinson’s disease, psoriasis, sleep apnea, and in specialist maternity care. These sixteen patient groups vary regarding their level of granularity with some referring to specific conditions and others spanning over several different diagnoses.

As described in the Model for Assessment of Telemedicine applications [14] assessment of telemedicine applications requires consideration of different aspects including the context and purpose of a telemedicine application, a multidisciplinary assessment of clinical effects but also patient perspectives, economic, organisational, ethical, legal and other aspects. The focus chosen in this overview is on the domains of safety and clinical effectiveness within this model for assessment.

As preparatory work for this overview, representatives at clinical departments working with the selected patient groups were interviewed. Thirteen of the sixteen departments intended to use remote patient monitoring to replace face-to-face visits offered in the current standard of care. In the remaining four clinical departments remote patient monitoring was considered an addition to standard of care.

The aim of this paper is *an overview and assessment of the methodological rigour of systematic reviews regarding remote patient monitoring, compared to standard of care, in selected patient groups*. The overview focuses patient-relevant clinical outcomes. Preparatory work for this overview was performed and published at the regional Health Technology Assessment (HTA) centre in Region Västra Götaland, Sweden [15].

## Methods

A comprehensive literature search and assessment of methodological rigour of systematic reviews (SRs) regarding remote patient monitoring compared to standard of care in the 16 selected patient groups, considered for remote patient monitoring in region Västra Götaland, was performed. Outcomes considered were clinical benefits as well as risks. The project was conducted by a team of clinical and methodological experts in Region Västra Götaland, Sweden.

Population, Intervention, Comparison, and Outcome (PICO) were defined as presented in the eligibility criteria in Table 1.

**Table 1:** Eligibility Criteria for the systematic reviews

	Inclusion	Exclusion
Population	Population belonging to one or more of the following patient groups: patients with asthma, chronic obstructive pulmonary disease, children and adolescents with complex needs, children and adolescents with cystic fibrosis, children and adolescents with periodic fever, elderly patients with multiple diseases, patients with eye diseases, heart failure, haematological disease, hypertension, inflammatory bowel disease, neuro-rehabilitation, Parkinson’s disease, psoriasis, sleep apnea, and specialist maternity care	Healthy population or patient population other than the selected patient groups
Intervention	Remote patient monitoring defined as: • Continuous follow-up of relevant health-related parameters of patients located outside healthcare facilities • Measurements are taken by analogue or digital devices, and objective and/or subjective assessments are delivered digitally to the patient and a healthcare unit • The healthcare unit provides the patient with feedback on the reported data (feedback may be automatically generated if data are within a predefined range) Remote patient monitoring may be offered as an add-on or replacement for standard of care	Other digital health interventions, such as: • Health-related parameters measured and registered by the patient without interaction with healthcare personnel • Remote interaction with healthcare personnel without continuous digital transfer of health-related parameters • Capturing health-related parameters for diagnostic purposes only
Comparator	No remote patient monitoring (standard of care)	
Outcomes	Patient-relevant, clinical benefits and/or risks	
Type of publication	Systematic review	

A search strategy was defined, and searches were conducted by two information specialists (TS and KM) in PubMed, Cochrane Library, The International Network of Agencies for Health Technology Assessment (INAHTA), and Swedish HTA websites in April 2022, with a limitation to SRs published from 2016 until April 2022. An updated search in PubMed was performed in February 2023. The search strategy is provided in Appendix 1. Independently, at least two project members assessed the relevance of identified abstracts and subsequently of full-text articles and decided in consensus on in- or exclusion of each publication based on the eligibility criteria provided in Table 1.

### Assessment of review methodology

**Fig. 1:**
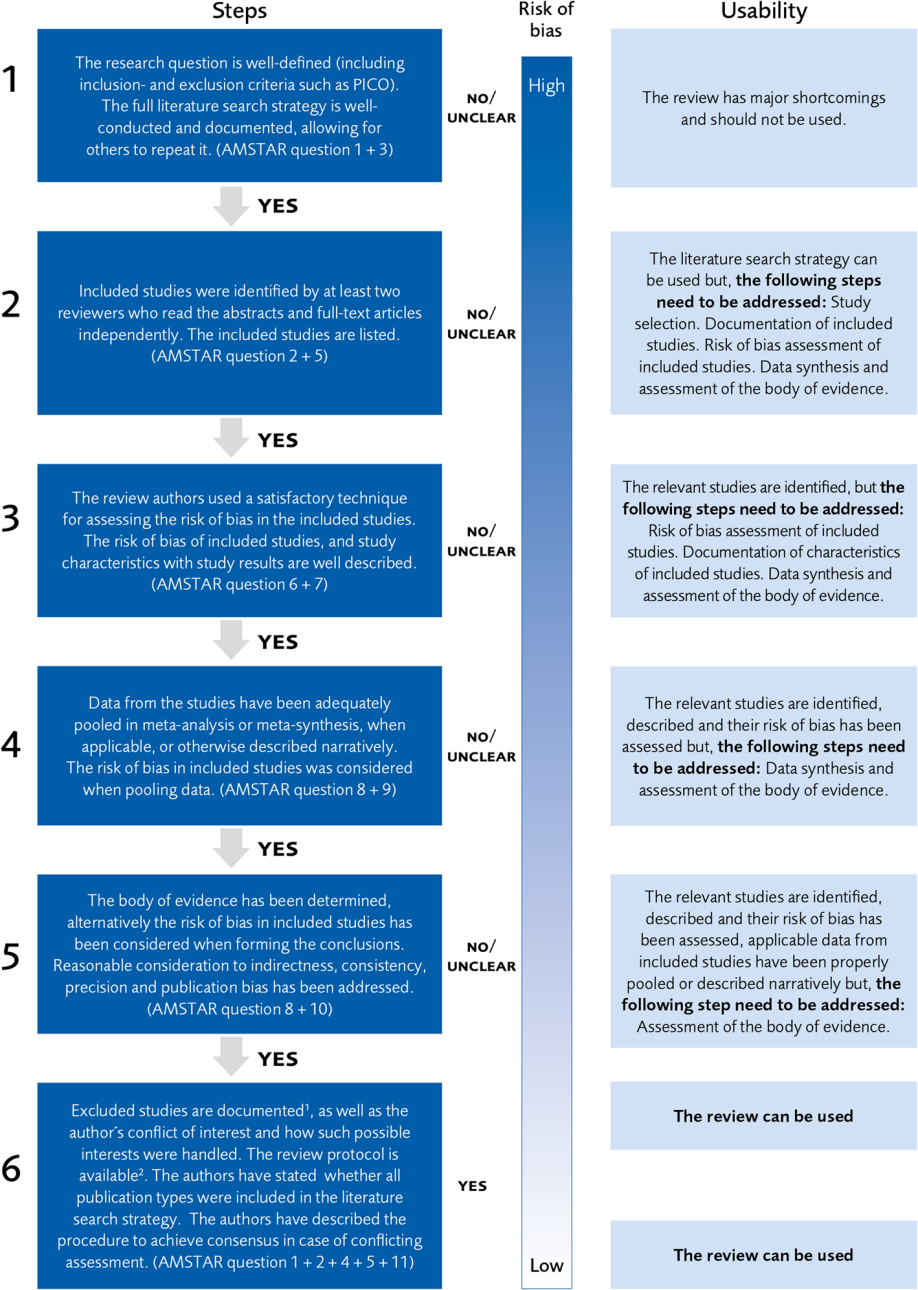
QUICKSTAR assessment tool. By courtesy of Swedish Agency for Health Technology Assessment and Assessment of Social Services (SBU). ^1.^The excluded studies ought to be listed in the review or in an appendix, or at least in a text that summarizes the reason for exclusions. However, this information may be missing due to limitations regarding the number of words that are allowed in some journals. SBU therefore finds that systematic reviews that do not fulfil this criterion may have moderate risk of bias and usability. ^2.^It is important that a protocol, which also aligns with the later published review, was set before conducting the systematic review. However, systematic reviews that were published some time ago seldom provide protocols, due to the traditions back then. Therefore, SBU finds that systematic reviews that do not fulfil this criterion have moderate usability

^1^The excluded studies ought to be listed in the review or in an appendix, or at least in a text that summarizes the reason for exclusions. However, this information may be missing due to limitations regarding the number of words that are allowed in some journals. SBU therefore finds that systematic reviews that do not fulfil this criterion may have moderate risk of bias and usability.

^2^It is important that a protocol, which also aligns with the later published review, was set before conducting the systematic review. However, systematic reviews that were published some time ago seldom provide protocols, due to the traditions back then. Therefore, SBU finds that systematic reviews that do not fulfil this criterion have moderate usability

The methodological rigour of included SRs was assessed independently by at least two project members (one with clinical background and one or two with expertise in health technology assessment) using QUICKSTAR, a tool for stepwise appraisal of SRs, developed by the Swedish Agency for Health Technology Assessment and Assessment of Social Services (SBU 2023) (see Fig. 1).

Based on AMSTAR-1 [16, 17], this tool implies a systematic assessment in six steps, as outlined in Fig. 1. The aspects that are appraised correspond to those evaluated in the AMSTAR-1 tool. The QUICKSTAR tool uses a stepwise assessment. Assessment with QUICKSTAR stops at the previous step if an SR does not fully meet the quality criteria of a step which offers an efficient approach to identifying SRs with sufficient quality. According to SBU who developed the tool, SRs not fulfilling the first step are considered not useful, and those reaching the fifth step and beyond can be used in health technology assessments with limited or no complementary work [18]. As shown in Fig. 1, the criterion of reaching the fifth step implies that essential quality criteria regarding the definition of the question at issue, the literature search, the risk of bias assessment, the adequacy of data synthesis, the systematic evaluation of the level of evidence, and adequate phrasing of the conclusion reflecting the level of evidence are met. After an independent assessment, project members reached a consensus on their assessment of each included SR and, if applicable, documented the rationale for stopping the QUICKSTAR process.

### Data extraction and synthesis

For each patient group, the number of SRs and the QUICKSTAR evaluation of these are provided. As reaching a QUICKSTAR level of at least five is considered necessary for reliable conclusions, detailed information is restricted to SRs reaching these levels. Data extraction for these SRs was limited to the number of included studies, the number of included randomised clinical trials (RCTs), the number of patients, and the overall conclusions as presented in the SR.

## Results

### Literature search

**Fig. 2:**
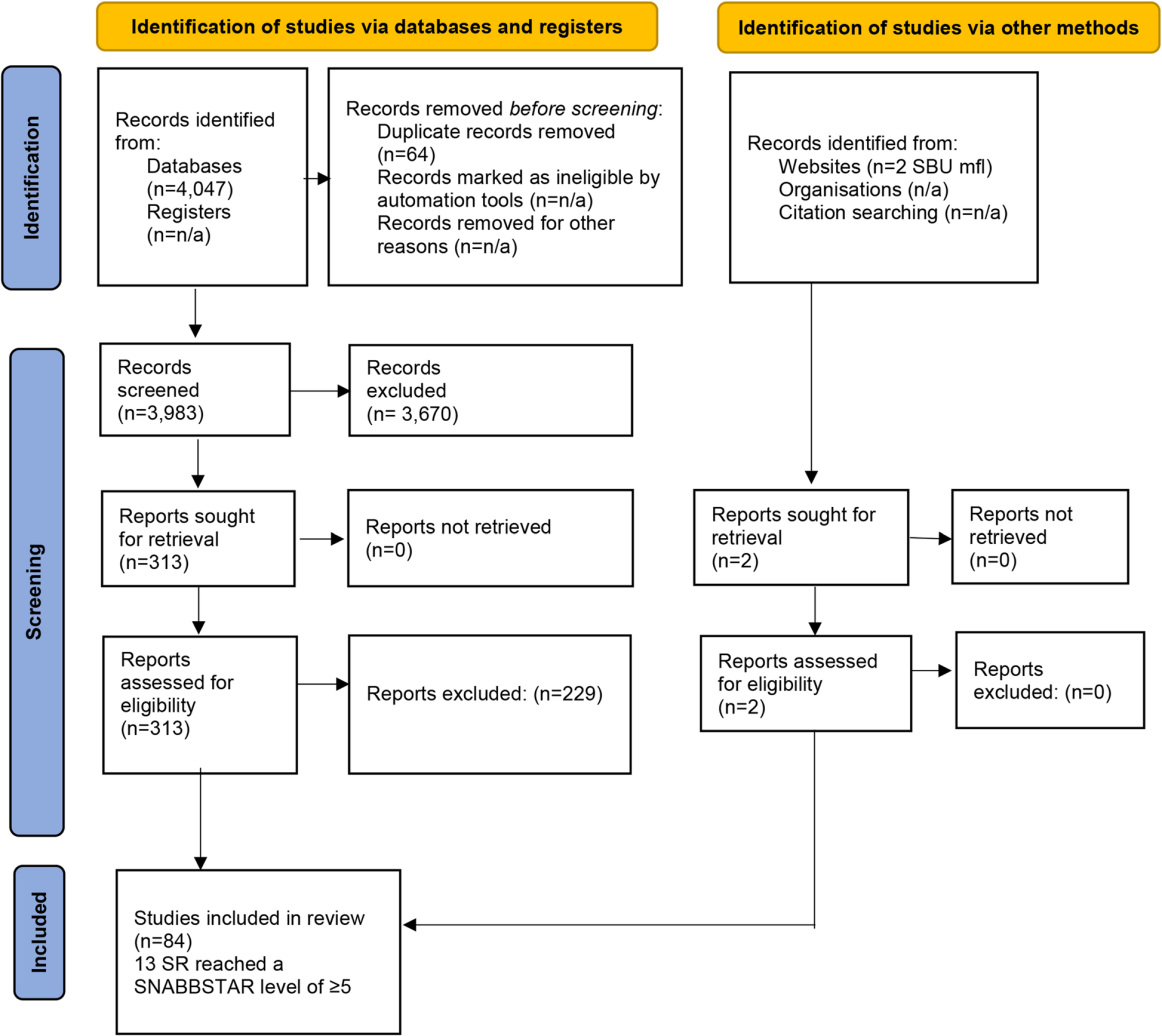
Prisma Flowchart, from Page MJ et al. [19]

As shown in Fig. 2, the literature search resulted in 4049 potentially relevant studies. Of these, 315 were read in full text to assess their relevance to our question (PICO). A total of 84 SRs were considered relevant, included, and subsequently assessed by QUICKSTAR (see Fig. 2 Prisma Flowchart).

### QUICKSTAR assessment

**Fig. 3:**
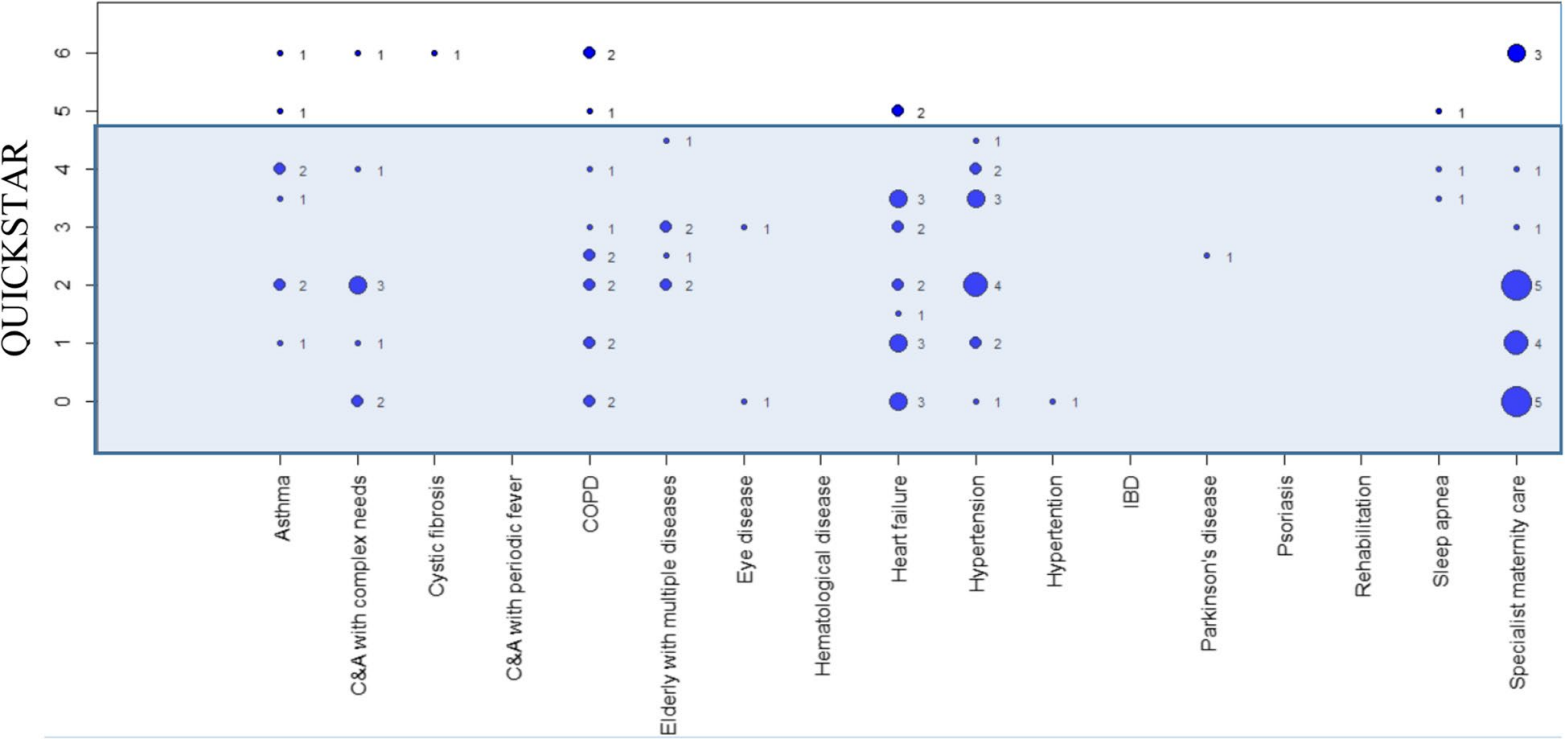
Number of SRs by patient group and QUICKSTAR level. C&A:Children and adolescents, IBD: Irritable Bowel Disease, COPD: Chronic obstructive pulmonary disease

Figure 3 shows the number of SRs reaching the different QUICKSTAR levels in the 16 patient groups. Of the included 84 SRs, 13 (15%) reached a QUICKSTAR level of at least 5. Table 2 presents for each patient group how many of the included SRs reached a QUICKSTAR of at least 5, and which overall conclusions were reported in these SRs. More detailed information on the assessment of included SRs is presented in Appendix 2 and characteristics of the SRs reaching a QUICKSTAR level of at least 5 are provided in Appendix 3.

**Table 2:** Summary by patient group – number of systematic reviews (SRs) included and number and overall conclusions of SRs reaching QUICKSTAR of at least 5

Patient group	Number of SRs reaching QUICKSTAR level ≥ 5^a^ / Number of included SRs	Overall conclusions in SRs reaching QUICKSTAR of at least 5 ^1^
Asthma	2/8^b^	Kew et al. [20] included 6 RCTs (2100 patients). They concluded that current evidence from randomised studies does not demonstrate any important differences between face-to-face and remote asthma check-ups in terms of exacerbations, asthma control or quality of life and that safety is insufficiently studied Nousios et al. [21] included 12 RCTs (351 patients). They stated that heterogeneous and biased studies reporting conflicting results prohibit any conclusion on the effect of using apps for remote monitoring and feedback in asthma
Children and adolescents with complex needs^2^	1/8	Thabrew et al. [22] included five RCTs (463 patients) of low quality regarding the treatment of depression/anxiety in populations with different diseases, concluding: Effects are uncertain, especially for children under ten years
Children and adolescents with cystic fibrosis	1/1	Toner et al. [23]did not identify any relevant RCTs regarding insulin therapy guided by continuous glucose monitoring systems in patients with cystic fibrosis-related diabetes
Children and adolescents with periodic fever	0/0	No relevant SR
Chronic obstructive lung disease	2/12^b^	Janjua et al. [24]: Remote monitoring as replacement: 10 RCTs (2456 patients) very low level of evidence Monitoring as add-on: Moderate evidence for remote monitoring reducing risk of COPD-related rehospitalisation (1 RCT (106 patients)). Very low level of evidence for other outcomes (8 RCTs, 1033 participants) Nousios et al. [21] included 6 RCTs (351 patients). They stated that heterogeneous and biased studies reporting conflicting results prohibit any conclusion on the effect of using apps for remote monitoring and feedback in COPD
Elderly patients with multiple diseases^3^	1/6	Kraef et al. [25] include 6 RCTs in populations with different combinations of diseases (699 patients) concluding that there is moderate evidence for improvement in measures of disease control, yet there may be little or no effect on patient-reported health status (low level of evidence)
Eye disease	0/2	No relevant SR reaching QUICKSTAR ≥ 5
Haematological disease	0/0	No relevant SR
Heart failure	2/16	Rebolledo et al. [26] included 19 RCTs (meta-analyses with up to 4375 patients) comparing monitoring (unclear whether as add-on or replacement) with usual care. They report a moderate level of evidence that monitoring reduces the risk of heart failure-related hospitalisation, non-statistically significant reduction in mortality risk, and variable results regarding different measures of Quality of Life Snellman et al. [27] including 13 RCTs (2636 patients) conclude: Heterogeneous material with very low level of evidence such that no conclusions on patient benefits and risks can be drawn
Hypertension	1/11^c^	Tucker et al. (2017), an individual patient data analysis with 15 RCTs (6300 patients), several with low risk of bias, concluding: On its own self-monitoring has no benefits, yet when combined with co-interventions leads to small but clinically relevant reduction in blood pressure
Inflammatory bowel disease	0/0	No relevant SR
Parkinson’s disease	0/1	No relevant SR reaching QUICKSTAR ≥ 5
Psoriasis	0/0	No relevant SR
Rehabilitation	0/0	No relevant SR
Sleep apnea	1/3	Murphie et al. [28] includes 5 studies (269 patients) concluding that there is very limited information and no definite evidence of effectiveness nor risks
Specialist maternity care	3/18^c^	Three SRs concluding that there is low or very low certainty of evidence Ashworth et al. [29] SR on pregnant women with hypertension (1 RCT, 154 patients) Moy et al. [30] SR on pregnant women with pre-existing type 2 diabetes mellitus (2 RCTs, 43 patients) Raman et al. [31] SR on pregnant women with pregnancy-related diabetes mellitus (5 RCTs, 478 patients)

*COPD* Chronic obstructive pulmonary disease, *RCT* Randomized Clinical Trial, *SR* Systematic review

^a^A QUICKSTAR level ≥ 5 was considered a precondition for reliable overall conclusions in SRs (conclusions phrased with consideration of the risk of bias of included studies and the level of evidence)

^b^One of these publications was relevant both for the patient group asthma and chronic obstructive lung disease

^c^One of these publications was relevant both for the patient group hypertension and specialist maternity care

In this figure, the size of the circle and the number to the right of the circle indicate the number of SRs for the respective patient group and QUICKSTAR level. A QUICKSTAR level of at least five (5) was considered a precondition for considering the overall conclusion of the SR to be reliable.

With 84 included SRs, the number of SRs for the selected patient groups was relatively high. However, SRs with a QUICKSTAR level of at least 5, a prerequisite for reliable conclusions, were only identified for nine of the 16 patient groups. In total, this level was reached by 13 (15%) of the included SRs. Only a few SRs (21 of 84) differentiated whether the analysis concerned using remote patient monitoring as a replacement or an addition to the standard of care.

In sum, some patient benefits of remote patient monitoring were reported in five patient groups (asthma, chronic obstructive lung disease, heart failure, hypertension, and elderly patients with multiple diseases). For four patient groups (children with complex needs, children with cystic fibrosis, specialist maternity care, and sleep apnea), systematic reviews of adequate quality concluded that scientific evidence on patient benefits of remote monitoring is very limited. For seven patient groups, no systematic reviews of sufficient quality were identified.

## Discussion

The aim of this paper was to provide an overview of the body of evidence regarding remote patient monitoring in 16 patient groups based on SRs. As reported in previous literature, remote patient monitoring has mainly focused on chronic conditions like diabetes, where it is expected to yield the most significant benefits [13]. It is often highlighted as enabling patient decision-making and self-care [11, 12], and as such, it is suggested as a potential solution for current challenges facing healthcare in terms of costs, quality and accessibility [6, 10]. Based on an overview of SRs, this study contributes to previous research by highlighting significant knowledge gaps regarding the benefits and risks of remote patient monitoring in the selected patient groups. Additionally, the overview underscores the need for methodological rigour when performing systematic reviews.

For the patient group asthma, two SRs were of adequate quality. One of them, Kew et al. [20], concluded that current evidence does not demonstrate any important differences between face-to-face and remote asthma check-ups in terms of exacerbations, asthma control or quality of life. However, the authors state that current evidence is insufficient to rule out differences in efficacy and that safety is insufficiently studied. The other SR on asthma, Nousios et al. [21] reaching QUICKSTAR level 5, was inconclusive due to the heterogeneity of the included studies.

For the three patient groups, hypertension, COPD and elderly patients with multiple diseases, some patient benefits from add-on remote patient monitoring were reported in the SRs, reaching a QUICKSTAR level of at least 5. For hypertension, an individual patient data analysis conducted by Tucker et al. [32] concluded that self-monitoring alone did not improve patients’ blood pressure. Yet they report that remote patient monitoring combined with co-interventions such as patient education or titration of medication according to professional advice, leads to clinically relevant reduction in blood pressure. For COPD, Janjua et al. [24] conclude that add-on remote patient monitoring reduced COPD-related re-admissions compared to standard of care alone (moderate level of evidence). Yet, for the other clinical outcomes evaluated in this SR (exacerbations, quality of life and mortality) and the comparison of remote patient monitoring with standard of care, the authors deemed the level of evidence as very low. Another SR on the use of mobile application for monitoring of COPD [21] was inconclusive due to the heterogeneity of the material. Regarding remote patient monitoring in elderly with multiple diseases, Kraef et al. [25] concluded that there is moderate evidence that remote patient monitoring, in addition to standard of care, probably improves disease control, e.g., blood pressure or diabetes control. However, they conclude that there may be little or no effect on patient-reported health status (low level of evidence).

In heart failure, Rebolledo et al. [26] reports moderate evidence that monitoring reduces the risk of heart failure-related hospitalisation, but findings that are not conclusive regarding mortality. This SR does not explicitly state whether monitoring was evaluated as a replacement or add-on to the standard of care. Snellman et al. [27] consider the material too heterogeneous to arrive at conclusions on patient benefits and risks.

For the other four patient groups for which SRs with a QUICKSTAR level of at least 5 were identified (children and adolescents with complex needs, children and adolescents with cystic fibrosis, specialist maternity care, and sleep apnea), the SRs stated that scientific information to evaluate patient benefits of remote patient monitoring is very limited and do not address the question whether remote monitoring is similar or better than standard of care. For the remaining seven patient groups (children and adolescents with periodic fever, haematological diseases, eye disorders, inflammatory bowel disease, Parkinson’s disease, psoriasis, and rehabilitation), no SR or none reaching QUICKSTAR level 5 was identified in this overview.

To sum up, based on an assessment with QUICKSTAR, a tool for assessment of the methodological rigour in SRs, we noted that only a few identified SRs in the field used established methods to provide reliable conclusions regarding the body of evidence. Most SRs that reach a QUICKSTAR level of 5 conclude that documentation of the benefits of remote patient monitoring added to the standard of care is limited. There is a shortage of reports on patient risks and evaluations of healthcare resource utilization costs. More studies and SRs utilizing established methodologies are required to address this gap. The findings from this study suggest a substantial need for well-designed studies examining the clinical benefits and risks for patients within these chosen patient groups. In future research, it is important to clearly differentiate whether remote patient monitoring is offered as a complement or replacement to conventional care, or whether a hybrid approach alternating remote and face-to-face care is used. In addition to addressing the gap identified in this paper regarding information on clinical benefits and risks, patient-reported outcomes and experiences are important to investigate in line with the model for assessment of telemedicine applications [14].

## Limitations

The present overview has several limitations. A key challenge is the generalizability of the results of the studies included in the SRs, and thus of the SRs as well as of the present overview. Remote patient monitoring can be provided in a wide variety of ways, e.g., with different selections of health-related parameters, modes and frequencies of measurement, frequency and method for reviewing patients’ data, procedures, and frequency of feedback to patients. Furthermore, the target population offered remote patient monitoring can differ between studies. Also, it is important to consider the healthcare context of usage: whether the method represents an additional procedure to complement conventional care or replace face-to-face visits in routine care. It is noted that remote patient monitoring may be used in a broad range of scenarios including hybrid approaches combining conventional face-to-face visits, with remote care by digital visits and remote patient monitoring. All these aspects challenge the generalizability of observations.

The present overview aimed to span several patient groups and was based on SRs rather than primary studies. Thus, this implies that the present overview is limited by the methodology and definitions of the included SRs, which often included studies with heterogeneous populations and or heterogeneous ways of remote patient monitoring. Furthermore, this overview does not reflect the most recent primary studies.

The scope of this overview was limited to clinical patient-relevant outcomes. Note, that in line with the model for assessment of telemedicine applications [14, 33] other aspects as patient satisfaction, healthcare professionals’ perspective, organizational and economic aspects are decisive factors to be considered when assessing and evaluating remote patient monitoring. These were out of scope for the present overview.

## Conclusion

For most patient groups considered in this overview, remote monitoring as a replacement for or as an addition to face-to-face visits compared to standard of care is poorly studied according to SRs using established evaluation methods. According to these reviews, patients’ clinical benefits are for most patient groups limited or impossible to evaluate based on currently available scientific information. Resource needs and possible clinical risks, especially for the scenario of remote monitoring replacing face-to-face visits in the standard of care are poorly studied in the SRs.

## Supplementary Information

Supplementary Material 1.

Supplementary Material 2.

Supplementary Material 3.

## Data Availability

All data generated or analysed during this study are included in this article and its Supplementary information files.

## Abbreviations

COPD: Chronic obstructive pulmonary disease
HTA: Health Technology Assessment
INAHTA: International Network of Agencies for Health Technology Assessment
IBD: Irritable Bowel Disease,
PICO: Population, Intervention, Comparison, and Outcome
RCT: Randomized Clinical Trial
SBU: Swedish Agency for Health Technology Assessment and Assessment of Social Services
SR: Systematic review
