# Supplementary material for: Clinical benefits and risks of remote patient monitoring: an overview and assessment of methodological rigour of systematic reviews for selected patient groups
Source: BMC Health Serv Res. 2025 Jan 23;25:133. doi: 10.1186/s12913-025-12292-w (PMC11759446; doi:10.1186/s12913-025-12292-w)
Supplement: Supplementary file 1 — Supplementary Material 1. [file 12913_2025_12292_MOESM1_ESM.docx]

**Appendix 1: Search Strategy**

**Database:** PubMed

**Date:** 19 April 2022
**Results:** 2,443

**Updated search:** 7 February 2023, 653 träffar

| **Search** | **Query** | **Results** |
| --- | --- | --- |
| **#13** | **Search: #3 NOT #6 Filters: Systematic Review, Danish, English, Norwegian, Swedish, from 2016 - 2022** | [**2,443**](https://pubmed.ncbi.nlm.nih.gov/?term=%233+NOT+%236&filter=pubt.systematicreview&filter=lang.danish&filter=lang.english&filter=lang.norwegian&filter=lang.swedish&filter=years.2016-2022&ac=no&sort=relevance) |
| #12 | Search: #3 NOT #6 Filters: Systematic Review, Danish, English, Norwegian, from 2016 - 2022 | [2,443](https://pubmed.ncbi.nlm.nih.gov/?term=%233+NOT+%236&filter=pubt.systematicreview&filter=lang.danish&filter=lang.english&filter=lang.norwegian&filter=years.2016-2022&ac=no&sort=relevance) |
| #8 | Search: #3 NOT #6 Filters: Systematic Review | [3,220](https://pubmed.ncbi.nlm.nih.gov/?term=%233+NOT+%236&filter=pubt.systematicreview&ac=no&sort=relevance) |
| #7 | Search: #3 NOT #6 | [141,107](https://pubmed.ncbi.nlm.nih.gov/?term=%233+NOT+%236&ac=no&sort=relevance) |
| #6 | Search: #4 OR #5 | [5,361,713](https://pubmed.ncbi.nlm.nih.gov/?term=%234+OR+%235&ac=no&sort=relevance) |
| #5 | Search: animal[ti] OR animals[ti] OR rat[ti] OR rats[ti] OR mouse[ti] OR mice[ti] OR rodent[ti] OR rodents[ti] OR dog[ti] OR dogs[ti] OR cat[ti] OR cats[ti] OR koalas[ti] OR hamster[ti] OR hamsters[ti] OR rabbit[ti] OR rabbits[ti] OR swine[ti] OR murine[ti] OR porcine[ti] | [2,009,984](https://pubmed.ncbi.nlm.nih.gov/?term=animal%5Bti%5D+OR+animals%5Bti%5D+OR+rat%5Bti%5D+OR+rats%5Bti%5D+OR+mouse%5Bti%5D+OR+mice%5Bti%5D+OR+rodent%5Bti%5D+OR+rodents%5Bti%5D+OR+dog%5Bti%5D+OR+dogs%5Bti%5D+OR+cat%5Bti%5D+OR+cats%5Bti%5D+OR+koalas%5Bti%5D+OR+hamster%5Bti%5D+OR+hamsters%5Bti%5D+OR+rabbit%5Bti%5D+OR+rabbits%5Bti%5D+OR+swine%5Bti%5D+OR+murine%5Bti%5D+OR+porcine%5Bti%5D&ac=no&sort=relevance) |
| #4 | Search: ((animals[mh]) NOT (animals[mh] AND humans[mh])) | [4,994,132](https://pubmed.ncbi.nlm.nih.gov/?term=%28%28animals%5Bmh%5D%29+NOT+%28animals%5Bmh%5D+AND+humans%5Bmh%5D%29%29&ac=no&sort=relevance) |
| #3 | Search: #1 OR #2 | [150,007](https://pubmed.ncbi.nlm.nih.gov/?term=%231+OR+%232&ac=no&sort=relevance) |
| #2 | Search: telemonitor*[Title/Abstract] OR tele-monitor*[Title/Abstract] OR homemonitor*[Title/Abstract] OR selfmonitor*[Title/Abstract] OR home-measured[Title/Abstract] OR home measurement*[Title/Abstract] OR home-management[Title/Abstract] OR self-monitor*[Title/Abstract] OR self-measured[Title/Abstract] OR self-measurement*[Title/Abstract] OR self-BPM[Title/Abstract] OR self blood pressure[Title/Abstract] OR home-BPM[Title/Abstract] OR home blood pressure*[Title/Abstract] OR telemedicine[Title/Abstract] OR tele-medicine[Title/Abstract] OR telehealth*[Title/Abstract] OR tele-health*[Title/Abstract] OR mhealth*[Title/Abstract] OR m-health*[Title/Abstract] OR ehealth*[Title/Abstract] OR e-health*[Title/Abstract] OR mobile health*[Title/Abstract] OR telemetr*[Title/Abstract] OR tele-metr*[Title/Abstract] OR ((monitor*[Title/Abstract]) AND (remote*[Title/Abstract] OR home[Title/Abstract] OR homebased[Title/Abstract] OR home-based[Title/Abstract] OR ambulatory[Title/Abstract] OR out-of-office[Title/Abstract] OR self-management[Title/Abstract])) | [112,555](https://pubmed.ncbi.nlm.nih.gov/?term=telemonitor%2A%5BTitle%2FAbstract%5D+OR+tele-monitor%2A%5BTitle%2FAbstract%5D+OR+homemonitor%2A%5BTitle%2FAbstract%5D+OR+selfmonitor%2A%5BTitle%2FAbstract%5D+OR+home-measured%5BTitle%2FAbstract%5D+OR+home+measurement%2A%5BTitle%2FAbstract%5D+OR+home-management%5BTitle%2FAbstract%5D+OR+self-monitor%2A%5BTitle%2FAbstract%5D+OR+self-measured%5BTitle%2FAbstract%5D+OR+self-measurement%2A%5BTitle%2FAbstract%5D+OR+self-BPM%5BTitle%2FAbstract%5D+OR+self+blood+pressure%5BTitle%2FAbstract%5D+OR+home-BPM%5BTitle%2FAbstract%5D+OR+home+blood+pressure%2A%5BTitle%2FAbstract%5D+OR+telemedicine%5BTitle%2FAbstract%5D+OR+tele-medicine%5BTitle%2FAbstract%5D+OR+telehealth%2A%5BTitle%2FAbstract%5D+OR+tele-health%2A%5BTitle%2FAbstract%5D+OR+mhealth%2A%5BTitle%2FAbstract%5D+OR+m-health%2A%5BTitle%2FAbstract%5D+OR+ehealth%2A%5BTitle%2FAbstract%5D+OR+e-health%2A%5BTitle%2FAbstract%5D+OR+mobile+health%2A%5BTitle%2FAbstract%5D+OR+telemetr%2A%5BTitle%2FAbstract%5D+OR+tele-metr%2A%5BTitle%2FAbstract%5D+OR+%28%28monitor%2A%5BTitle%2FAbstract%5D%29+AND+%28remote%2A%5BTitle%2FAbstract%5D+OR+home%5BTitle%2FAbstract%5D+OR+homebased%5BTitle%2FAbstract%5D+OR+home-based%5BTitle%2FAbstract%5D+OR+ambulatory%5BTitle%2FAbstract%5D+OR+out-of-office%5BTitle%2FAbstract%5D+OR+self-management%5BTitle%2FAbstract%5D%29%29&ac=no&sort=relevance) |
| #1 | Search: "Monitoring, Ambulatory"[Mesh] OR "Telemedicine"[Mesh:NoExp] OR "Telemetry"[Mesh] | [75,227](https://pubmed.ncbi.nlm.nih.gov/?term=%22Monitoring%2C+Ambulatory%22%5BMesh%5D+OR+%22Telemedicine%22%5BMesh%3ANoExp%5D+OR+%22Telemetry%22%5BMesh%5D&ac=no&sort=relevance) |

**Database:** The Cochrane Library (Wiley)
**Date:** 19 April 2022
**Results:** 75

*Cochrane reviews: 75*

| **ID** | **Search** | **Results** |
| --- | --- | --- |
| #1 | MeSH descriptor: [Monitoring, Ambulatory] explode all trees | 3325 |
| #2 | MeSH descriptor: [Telemedicine] this term only | 2657 |
| #3 | MeSH descriptor: [Telemetry] explode all trees | 306 |
| #4 | (telemonitor* OR (tele NEXT monitor*) OR homemonitor* OR selfmonitor* OR (home NEXT measured) OR (home NEXT measurement*) OR (home NEXT management) OR (self NEXT monitor*) OR (self NEXT measured) OR (self NEXT measurement*) OR (self NEXT BPM) OR "self blood pressure" OR (home NEXT BPM) OR "home blood pressure" OR "home blood pressures" OR telemedicine OR (tele NEXT medicine) OR telehealth* OR (tele NEXT health*) OR mhealth* OR (m NEXT health*) OR ehealth* OR (e NEXT health*) OR (mobile NEXT health*) OR telemetr* OR (tele NEXT metr*) OR ((monitor*) AND (remote* OR home OR homebased OR (home NEXT based) OR ambulatory OR (out NEXT of NEXT office) OR (self NEXT management)))):ti,ab,kw (Word variations have been searched) | 29953 |
| #5 | #1 OR #2 OR #3 OR #4 | 30454 |
| **Limit search to Cochrane Reviews and publication year 2016-2022** | | **75** |

**Database:** International HTA database (Inatha)
**Date:** 19 April 2022
**Results:** 876

| telemonitor* OR (tele monitor*) OR homemonitor* OR selfmonitor* OR (home measured) OR (home measurement*) OR (home management) OR (self monitor*) OR (self measured) OR (self measurement*) OR (self BPM) OR (self blood pressure) OR (home BPM) OR (home blood pressure*) OR telemedicine OR (tele medicine) OR telehealth* OR (tele health*) OR mhealth* OR (m health*) OR ehealth* OR (e health*) OR (mobile health*) OR telemetr* OR (tele metr*) |  |
| --- | --- |
| telemonitor* OR (tele monitor*) OR homemonitor* OR selfmonitor* OR (home measured) OR (home measurement*) OR (home management) OR (self monitor*) OR (self measured) OR (self measurement*) OR (self BPM) OR (self blood pressure) OR (home BPM) OR (home blood pressure*) OR telemedicine OR (tele medicine) OR telehealth* OR (tele health*) OR mhealth* OR (m health*) OR ehealth* OR (e health*) OR (mobile health*) OR telemetr* OR (tele metr*) |  |
| OR |  |
| ((monitor*) AND (remote* OR home OR homebased OR (home-based) OR ambulatory OR (out-of-office) OR (self-management))) |  |
| **Limit search to english, swedish, danish, norwegian and publication year 2016-2022** | **876** |

*(The above keywords were searched in title, abstract, and keywords)*
