# Supplementary material for: Clinical benefits and risks of remote patient monitoring: an overview and assessment of methodological rigour of systematic reviews for selected patient groups
Source: BMC Health Serv Res. 2025 Jan 23;25:133. doi: 10.1186/s12913-025-12292-w (PMC11759446; doi:10.1186/s12913-025-12292-w)
Supplement: Supplementary file 2 — Supplementary Material 2. [file 12913_2025_12292_MOESM2_ESM.docx]

**Appendix 2: QUICKSTAR assessment of systematic reviews**

| **Publication** | **Level1:** Question | **Level 2:**  Assessment of relevance | **Level 3:** Risk of bias assessment | **Level 4:**  Meta-analys /narrativ syntes | **Level 5:**  Grading of evidence | **Level 6:** Exclusions, statement of conflict of interest, protocol | **Comment** | **Differenti-ation remote monitoring as add on or replacement of SoC** |
| --- | --- | --- | --- | --- | --- | --- | --- | --- |
| **Asthma** | | | | | | | |  |
| Hodkinson et al., 2020, UK | √ | √ | √ | √ |  |  | Unclear which aspects were considered in evidence grading | Yes (as outcome) |
| Hui et al., 2017,  UK | √ |  |  |  |  |  | Relevance not assessed by two independent reviewers | Unclear |
| Jeminiwa et al.,2019,  USA | ? | √ | √ | ? |  |  | Unclear consideration of RoB in meta-analyses, No grading of evidence | Unclear |
| Kew et al., 2016,  UK | √ | √ | √ | √ | √ | √ |  | Yes (monitoring as add on) |
| McLean et al., 2016, UK | √ | √ |  |  |  |  | Incomplete Cochrane RoB assessment | Unclear |
| Nousios et al., 2022, Sweden | √ | √ | √ | √ | √ |  | No published protocol | Unclear |
| Schulte et al., 2021, Netherlands | √ | √ | √ | ? |  |  | Results only described, no evidence grading | Unclear |
| Snoswell et al., 2021, Australia | √ | √ |  |  |  |  | Incomplete RoB assessment | Unclear |
| **Children and adolescents with complex needs** | | | | | | | |  |
| Badawy et al., 2017, USA | √ | √ |  |  |  |  | Incomplete RoB description | Unclear |
| Crawford et al., 2021,  UK | √ | ? |  |  |  |  | Screening by one reviewer only | Unclear (some information on healthcare use as outcome) |
| De Guzman et al., 2020, Australia | √ | √ |  |  |  |  | RoB assessment not provided | Unclear |
| Karatas et al., 2022, Turkey | √ | ? | √ | ? |  |  | Unclear whether relevance assessment by two reviewers,  Unclear grading of evidence and how RoB was considered in that | Unclear |
| Knox et al., 2019,  UK | √ |  |  |  |  |  | Screening by one reviewer only | Yes (add on) |
| Rodriguez et al., 2021, Spain |  |  |  |  |  |  | Incomplete information on the search strategy | Unclear |
| Sasangohar et al., 2018, USA |  |  |  |  |  |  | Incomplete information on the search strategy | Unclear |
| Thabrew et al., 2018, New Zealand | √ | √ | √ | √ | √ | √ |  | Unclear (no data on the outcome - number of visits) |
| **Children and adolescents with cystic fibrosis** | | | | | | | |  |
| Toner et al., 2021,  UK | √ | √ | √ | √ | √ | √ |  | Yes, yet no studies found |
| **COPD** | | | | | | | |  |
| Al Rajeh et al., 2016,  UK | √ |  |  |  |  |  | No description of study design or control groups | Unclear |
| Baroi et al., 2018,  Australia | √ | √ | ? |  |  |  | RoB assessment not according to established method | Unclear |
| Gaveikaite et al., 2019, Greece | ? | ? |  |  |  |  | Selection based on previous review paper, unclear inclusion, RoB assessment not described | Yes |
| Hong et al., 2019,  Republic of Korea | √ | √ | ? |  |  |  | Unclear assessment of RoB regarding blindning, Many meta-analyses without consideration of RoB or multiplicity | Unclear |
| Jang et al., 2021,  Republic of Korea | √ | ? | √ |  |  |  | Meta-analyses of heterogeneous material | Unclear |
| Janjua et al., 2021,  UK | √ | ? | √ | √ | √ | √ | Some limitations in information on included studies (eg type of devices, frequency of monitoring, definition of outcomes etc) | Yes |
| Lu et al., 2021,  China | √ |  |  |  |  |  | Unclear selection of studies | Yes (add on) |
| Nagase et al, 2022 | √ | √ | √ | √ |  |  | Lack of clarity in assessment of the body of evidence and consideration of this in the conclusions | Yes (number of visits as outcome) |
| Nousios et al, 2022,  Sweden | √ | √ | √ | √ | √ |  | Note – limited to mobile applications.  No published protocol | Unclear |
| Sul et al., 2020,  Republic of Korea |  |  |  |  |  |  | Unclear definition of primary outcome – every interaction with COPD caregivers is counted as an exacerbation | Unclear |
| Taylor et al., 2021,  Australia | √ | √ |  |  |  |  | RoB assessments not provided | Unclear |
| Yang et al., 2017,  USA |  |  |  |  |  |  | Lack of information on search strategy | Unclear |
| **Elderly patients with multiple diseases** | | | | | | | |  |
| Dennett et al., 2021,  UK | √ | √ | √ |  |  |  | Meta-analyses of heterogeneous material (eg. studies with mere telemonitoring and comprehensive rehabilitation interventions) | Unclear |
| Kirakalaprathapan et al., 2022, Canada | √ | √ | √ |  |  |  | Unclear presentation of meta-analyses, (line of unity not marked in forest plots, CI not considered in result interpretation) | Unclear |
| Kraef et al., 2020,  Germany | √ | √ | √ | ? | ? |  | Limited consideration of RoB in grading of evidence,  Excluded studies not listed | Yes (descriptive) |
| Linn et al., 2021, Luxembourg | √ | √ | ? |  |  |  | Unclear which studies were assessed regarding RoB, result presentation with focus on positive results. (Note – scoping review) | Unclear |
| Liu et al., 2016,  Canada | √ | √ |  |  |  |  | No clear presentation of RoB assessments | Unclear |
| Liu et al., 2019,  China | √ | √ |  |  |  |  | No clear presentation of RoB assessments | Unclear |
| **Eye diseases** | | | | | | | |  |
| Ittoop et al., 2016, USA |  |  |  |  |  |  | Search not documented | Unclear |
| Tseng et al., 2021, Singapore | √ | √ | √ |  |  |  | No consideration of RoB in presentation of meta-analyses | Unclear |
| **Heart failure** | | | | | | | |  |
| Aronow et al., 2018,  USA | ? |  |  |  |  |  | Search terms not provided | Unclear |
| Auener et al., 2021,  Netherlands | √ | √ | ? |  |  |  | Limited information on RoB assessment, Limited quantitative information from individual studies | Yes (healthcare utilisation as outcome) |
| Carbo et al., 2018,  USA | ? |  |  |  |  |  | Limited information on search terms, limited information on included studies | Unclear |
| Clark et al., 2018,  Australia |  |  |  |  |  |  | Limited information on literature search | Unclear |
| De Guzman et al., 2022, Australia | √ | √ |  |  |  |  | Systematic review of economic evaluations – no risk of bias assessment | Yes (as outcome in economic analysis) |
| Ding et al., 2020,  Australia | √ | √ | √ |  |  |  | RoB not considered in meta-analyses, presentation with focus on significant results | Unclear |
| Drews et al., 2021,  Finland | √ |  |  |  |  |  | Only for a subset of articles relevance was assessed by two reviewers | Unclear |
| Kitsiou et al., 2021,  USA | √ | √ | √ | ? |  |  | Meta-analysis of clinically heterogeneous material, RoB not considered in conclusions | Unclear |
| Lin et al., 2017,  China | √ | √ | √ | ? |  |  | Meta-analysis of clinically heterogeneous material, RoB not considered in conclusions | Unclear |
| Liu et al., 2022, China | √ | ? | √ |  |  |  | Unclear whether relevance was assessed by two reviewers. Meta-analysis of studies with different interventions using fixed effects method. | Unclear |
| Pekmezaris et al., 2018, USA | √ |  |  |  |  |  | No flow chart, key studies in the field are missing | Unclear |
| Rebolledo del Toro et al., 2023, Colombia | ? | √ | √ | √ | ? |  | Few hits compared to other reviews.  Overall conclusion does not explicitly express the uncertainty (GRADE level), and some unclarity regarding GRADE for QoL.  No list of excluded studies | Unclear |
| Umeh et al., 2022, USA |  |  |  |  |  |  | Comparator not specified, and limited information on search strategy | Unclear |
| Yanicelli et al., 2021,  Argentine | √ |  |  |  |  |  | No flow chart in/exclusion by one reviewer only | Unclear |
| Yun et al., 2018,  Republic of Korea | √ | √ |  |  |  |  | Unclear RoB assessment | Unclear |
| Snellman et al., 2022,  Sweden | √ | √ | √ | √ | ? |  | Very strict judgement of the material, no published protocol | Unclear |
| **Hypertension** | | | | | | | |  |
| Blok et al., 2021,  Netherlands | √ | √ | √ | ? |  |  | Meta-analyses of clinically heterogeneous material | Yes |
| Choi et al., 2021,  Republic of Korea | √ | √ |  |  |  |  | No information on RoB assessment per study | Yes (add on) |
| Kalafat et al., 2020,  Turkey | √ | √ |  |  |  |  | Incomplete information on RoB assessments | Unclear |
| Kaihara et al., 2022,  Japan | √ | √ | √ | √ |  |  | No assessment of the body of evidence | Unclear |
| Kalagara et al., 2022, USA | √ |  |  |  |  |  | Relevance nor assessed by two independent reviewers | Unclear |
| Khanijahani et al., 2022, USA | √ | √ |  |  |  |  | No RoB assessment | Unclear |
| Li et al., 2020,  UK | √ | √ | √ | ? |  |  | Combination of studies without consideration of RoB and unclarity in meta-analyses | Unclear |
| Ma et al., 2022, China |  |  |  |  |  |  | Lack of clarity regarding the literature search | Unclear |
| Satoh et al., 2019,  Japan | √ | √ | √ | ? |  |  | Meta-analyses of clinically heterogeneous material | Unclear |
| Sheppard et al., 2020, UK | √ | √ |  |  |  |  | No information on RoB assessment per study | Yes |
| Tucker et al., 2017,  USA | √ | √ | √ | √ | ? |  | Conclusions not worded in line with evidence GRADE. | Yes |
| **Parkinson’s disease** | | | | | | | |  |
| Lee et al., 2022,  Republic of Korea | √ | √ | ? |  |  |  | Unclear RoB assessment, limited provision of results | Unclear |
| **Sleep apnea** | | | | | | | |  |
| Labarca et al., 2021,  USA | ? | **√** | **√** | ? |  |  | Some unclarity on comparator, meta-analyses of clinically heterogeneous material, conclusions without consideration of evidence level | Unclear |
| Murphie et al., 2019,  UK | **√** | **√** | **√** | **√** | **√** |  | No list of excluded studies | Unclear |
| Patil et al., 2019,  USA | **√** | **√** | ? | ? |  |  | No information on RoB assessment per study, meta-analysis of and conclusions on heterogeneous material without consideration of RoB | Unclear |
| **Specialist maternity care** | | | | | | | |  |
| Alves et al., 2020,  Brazil |  |  |  |  |  |  | Incomplete description on search strategy | Unclear |
| Ashworth et al., 2020, UK | **√** | **√** | ? | **√** | **√** | **√** | Unclear assessment of RoB regarding blinding | Yes |
| Bertini et al., 2022,  Chile |  |  |  |  |  |  | Incomplete description on search strategy | Unclear |
| Eberle et al., 2021a,  Germany | **√** | **√** |  |  |  |  | Incomplete assessment of RoB | Unclear |
| Eberle et al., 2021b,  Germany | **√** |  |  |  |  |  | Unclear whether relevance assessed by two independent reviewers | Yes |
| Eberle et al., 2021c,  Germany | **√** |  |  |  |  |  | Unclear whether relevance assessed by two independent reviewers | Unclear |
| Fantinelli et al., 2019,  Italy | **√** |  |  |  |  |  | Unclear whether relevance assessed by two independent reviewers | Unclear |
| Garg et al., 2022,  India |  |  |  |  |  |  | Incomplete description on search strategy | Unclear |
| HTA Wales, 2019,  UK |  |  |  |  |  |  | Incomplete description on search strategy | Unclear |
| HTA Wales, 2020,  UK |  |  |  |  |  |  | Incomplete description on search strategy | Unclear |
| Kalafat et al., 2020,  India | **√** | **√** |  |  |  |  | Unclear RoB assessment | Yes (outcome antenatal visits) |
| Lau et al., 2016,  Singapore | **√** | **√** |  |  |  |  | Unclear RoB assessment | Unclear |
| Laurensen et al., 2022, Denmark | **√** | ? | ? |  |  |  | Limited information on included studies, unclear assessment of RoB regarding blinding, meta-analyses of heterogeneous material, numerous analyses without consideration of multiplicity | Unclear |
| Ming et al., 2016,  UK | **√** |  |  |  |  |  | Unclear whether relevance assessed by two independent reviewers | Unclear |
| Moy et al., 2017,  Malaysia | **√** | **√** | ? | **√** | **√** | **√** | Some unclarity in RoB assessment regarding blinding | Yes |
| Raman et al., 2017,  Australia | **√** | **√** | **√** | **√** | **√** | **√** |  | Yes, (as outcome) |
| Urquhart et al., 2017,  UK | **√** | **√** | **√** | **√** |  |  | Assessment as high evidence level although unclear results in primary and sensitivity analyses | Yes |
| Xie et al., 2020,  China | **√** | **√** |  |  |  |  | Unclear RoB assessment | Unclear |
