# Supplementary material for: Clinical benefits and risks of remote patient monitoring: an overview and assessment of methodological rigour of systematic reviews for selected patient groups
Source: BMC Health Serv Res. 2025 Jan 23;25:133. doi: 10.1186/s12913-025-12292-w (PMC11759446; doi:10.1186/s12913-025-12292-w)
Supplement: Supplementary file 3 — Supplementary Material 3. [file 12913_2025_12292_MOESM3_ESM.docx]

**Appendix 3:** Characteristics of systematic reviews (SRs) reaching QUICKSTAR of at least 5

| **Systematic review Author**  **Country** | **QUICKSTAR level** | **Population** | **Intervention (I)**  **/Comparator (C)** | **Clinical outcome** | **Number of included studies (RCTs)** | **Number of patients** | **Conclusion according to the publication (Quote)** |
| --- | --- | --- | --- | --- | --- | --- | --- |
| **Asthma** | | | | | | | |
| Kew 2016  Great Britain | 6 | Adults and children with asthma | Monitoring in between physical visits | - Asthma exacerbation requiring cortison treatment - HrQoL - Lung function - Adverse events | 6 (6) | 2100 | “Current randomised evidence does not demonstrate any important differences between face-to-face and remote asthma check-ups in terms of exacerbations, asthma control or quality of life. There is insufficient information to rule out differences in efficacy, or to say whether or not remote asthma check-ups are a safe alternative to being seen face-to-face" |
| Nousios 2022  Sweden | 5 | Patients over 12 years of age with asthma (the report also includes a separate analysis on patients with COPD) | Mobile phone applications transferring health data to healthcare unit for assessment and feedback | - Mortality - Exacerbations - Lung function - Treatment compliance - Adverse events - Anxiety - Resource use - Costs, - HrQoL | 12 (12) | 1769 | “Heterogeneous and biased studies reporting conflicting results prohibit any conclusion on the effect of using apps for remote monitoring and feedback in asthma and COPD as compared with usual care.” (Corresponding conclusion regarding patients with COPD.) |
| **Children with complex needs** | | | | | | | |
| Thabrew, 2018  New Zealand | 6 | Children, 10–18 years of age, with chronic somatic disease (asthma, headache, migrane, cystic fibrosis, musculosceletal pain etc). | I: E-health interventions with remote patient monitoring, (including eg patient diaries shared with healthcare)  C: Standard of care | - Depression - Anxiety - HrQoL - Everyday functioning, - Long-term somatic health | 5 (5) | 463 | “At present, the field of e-health interventions for the treatment of anxiety or depression in children and adolescents with long-term physical conditions is limited to five low quality trials. The very low-quality of the evidence means the effects of e-health interventions are uncertain at this time, especially in children aged under 10 years.” |
| **COPD** | | | | | | | |
| Janjua  2021  Great Britain | 6 | Patients with COPD | I1: Remote patient monitoring added to standard of care,  I2: Remote patient monitoring  C: Standard of care | - Exacerbations - HrQoL - Dyspnea symptoms - Use of hospital care - Mortality | I1: 8 (8)  I2: 10 (10) | I1: 1033  I2: 2456 | “Remote monitoring plus usual care provided asynchronously may not be beneficial overall compared to usual care alone. Some benefit is seen in reduction of COPD-related hospital re-admissions, but moderate-certainty evidence is based on one study. We have not found any evidence for dyspnoea symptoms nor harms, and there is no difference in fatalities when remote monitoring is provided in addition to usual care.  Remote monitoring interventions alone are no better than usual care overall for health outcomes.” |
| Nousios 2022  Sweden | 5 | Patients over 12 years of age with COPD (the report also provides a separate analysis regarding patients with asthma) | Mobile phone applications transfering health data to healthcare unit for assessment and feedback | - Mortality - Exacerbations - Lung function, - Treatment compliance - Adverse events - Anxiety - Resource use - Costs, - HrQoL | 6 (6) | 1705 | “Heterogeneous and biased studies reporting conflicting results prohibit any conclusion on the effect of using apps for remote monitoring and feedback in asthma and COPD as compared with usual care.” (Corresponding conclusion regarding patients with asthma.) |
| **Cystic fibrosis** | | | | | | | |
| Toner 2021  Great Britain | 6 | Patients with cystic fibrosis related diabetes | I: Insulin treatment using online system för continuous glucose monitoring glukosmonitorering  C: Insulin treatment with other systems for glucose monitoring | - HrQoL - Glycemic control | 0 | 0 | ” No studies were included in the review, indicating that there is currently insufficient evidence to determine the impact of insulin therapy guided by CGMS compared to insulin therapy guided by other forms of glucose data collection on the lives of people with CFRD, nor on potential adverse effects of continuous glucose monitoring in this context. Randomised controlled studies are needed to generate evidence on the efficacy and safety of continuous glucose monitoring in people with CFRD. There is one relevant ongoing study that may be eligible for inclusion in a future update of this Cochrane Review, and whose results may help answer the review question.” |
| **Elderly patients with multiple diseases^3^** | | | | | | | |
| Kraef 2020  Germany | 4-5 | Patients with multiple diseases | Telemonitoring of blood pressure, glucose levels, physical activity, combined with individualized automated feedback and lifestyle advice  Note, one of six studies (Donesky 2017) with 15 patients concerned videobased telecare only. | - Change in systolic blood pressure, HbA1C, total cholesterol - Mental health - HrQoL - Hospitalisation | 6 | 699 | “Our systematic review shows that evidence for the effectiveness of digital telemedicine interventions for multimorbidity is very limited.”  “Digital telemedicine interventions provided moderate evidence of improvements in measures of disease control but little evidence and no demonstrated benefits on health status.”  NOTE: The population was not limited to specific years of age |
| **Heart failure** | | | | | | | |
| Snellman 2022  Sweden | 4-5 | Patients (aged 12 years or more) with chronic heartfailure managed in primary or specialist healthcare. | I: patient sends health data by mobile app to healthcare for assessment. Healthcare provides feedback to the patient.  C: standard of care | - Mortality - hospitalization - duration of care, - HrQoL | 13 (13) | 2636 | “Heterogenous and biased studies reporting non-significant or contradictory results prohobited any conclusion on the effect of using health apps for remote monitoring and feedback for patients with HF.” |
| Rebodello 2023  Colombia | 4-5 | Patients with heartfailure | I: Remote patient monitoring (unclear whether as add on or replacement)  C: Usual care | - Mortality - Hospitalization - HrQoL | 19 (19) | 4375 | “Non-statistically signifcant reduction in mortality risk was observed. The impact on QoL was variable between studies, with diferent scores and reporting measures used, thus limiting data pooling. The use of mobile-based telemonitoring strategies in patients with HF reduces risk of hospitalization due to HF. As smartphones and wirelessly connected devices are increasingly available, further research on this topic is warranted, particularly in the foundational therapy.” |
| **Specialist maternity care** | | | | | | | |
| Ashworth 2020  Great Britain | 6 | Pregnant women with chronic or pregnancy-related hypertension | I: Monitoring of home blood pressure measurements  C: Standard of care | Mother:   - Mortality - Pre-eclampsia - Intensive care - Labor induction   Child:   - Mortality - Preterm birth - Intensive care | 1 (1) | 154 | “The benefit, if any, of self-monitoring BP in hypertensive pregnancies remains uncertain, as the evidence is limited to one feasibility study.” |
| Moy 2017  Malaysia | 6 | Pregnant women with Type1 or Type 2 diabetes mellitus before pregnancy | I: Telemedicine  C: Standard of care | - Cesarian section - Glykemic control - Perinatal mortality | 2 (2) | 43 | “This review found no evidence that any glucose monitoring technique is superior to any other technique among pregnant women with pre-existing type 1 or type 2 diabetes. The evidence base for the effectiveness of monitoring techniques is weak and additional evidence from large well-designed randomised trials is required to inform choices of glucose monitoring techniques.”  (Note the publication includes 4 comparisons one of which is relevant for this report.) |
| Raman 2017  Australia | 6 | Pregnant women with pregnancy-related diabetes mellitus | I: Transfer of glucose measurements from patients’ home to the healthcare unit  C: standard of care | Mother:   - Pregnancy related hypertension - Cesarian section - Development of Type 2 Diabetes Mellitus - Labor induction   Child:   - Perinatal mortality - Macrosomia - Hypoglycaemia - Composite (mortality or severe morbidity) | 5 (5) | 478 | “Evidence from 11 RCTs assessing different methods or settings for glucose monitoring for GDM [gestational diabetes mellitus] suggests no clear differences for the primary outcomes or other secondary outcomes assessed in this review. However, current evidence is limited by the small number of RCTs for the comparisons assessed, small sample sizes, and the variable methodological quality of the RCTs.“  (Notera att publikationen avser 5 jämförelser varav enbart en (med 5 RCT som underlag) är relevant för vår frågeställning). |
| **Sleep Apnea** | | | | | | | |
| Murphie 2019  Great Britain | 5 | Patients with obstructive sleep apnea treated with Continuous positive airway pressure | I: Telemonitoring and teleconsultation C: Standard of care | - Treatment compliance - Sleepiness (Epworth sleepiness scale) - Patient satisfaction - Costs | 5 (4) | 269 | “The evidence base for the effectiveness of remote consultations with telemonitoring in the clinical review of those using CPAP therapy is limited to two small studies and three larger studies four of which were at moderate  to high risk of bias, and one at moderate risk of bias. These studies do not provide definitive evidence of effectiveness (in terms of adherence and symptom control) of teleconsultation/ telemonitoring in CPAP users;  however, there is no suggestion of any harms. “ |

**^a^** A QUICKSTAR level ≥5 was considered a precondition for reliable overall conclusions in SRs (conclusions phrased with consideration of the risk of bias of included studies and the level of evidence).

COPD: Chronic obstructive pulmonary disease, HRQoL: Health related quality of life, RCT: Randomized Clinical Trial, SR: Systematic review
